# Rice *Xa21* primed genes and pathways that are critical for combating bacterial blight infection

**DOI:** 10.1038/srep12165

**Published:** 2015-07-17

**Authors:** Hai Peng, Zheng Chen, Zhiwei Fang, Junfei Zhou, Zhihui Xia, Lifen Gao, Lihong Chen, Lili Li, Tiantian Li, Wenxue Zhai, Weixiong Zhang

**Affiliations:** 1 Institute for Systems Biology, Jianghan University, Wuhan, Hubei 430056, China; 2 Department of Computer Science and Engineering, Washington University in St. Louis, St. Louis, MO 63130, USA; 3 Hainan Key Laboratory for Sustainable Utilization of Tropical Bioresources, Institute of BioScience and Technology, College of Agriculture, Hainan University, Haikou 570228, China; 4 Institute of Genetics and Developmental Biology, Chinese Academy of Science, Beijing 100101, China; 5 Department of Genetics, Washington University in St. Louis, St. Louis, MO, 63130, USA

## Abstract

Rice bacterial blight (BB) is a devastating rice disease. The *Xa21* gene confers a broad and persistent resistance against BB. We introduced *Xa21* into *Oryza sativa* L ssp *indica* (rice 9311), through multi-generation backcrossing, and generated a nearly isogenic, blight-resistant 9311/*Xa21* rice. Using next-generation sequencing, we profiled the transcriptomes of both varieties before and within four days after infection of bacterium *Xanthomonas oryzae* pv. *oryzae*. The identified differentially expressed (DE) genes and signaling pathways revealed insights into the functions of *Xa21*. Surprisingly, before infection 1,889 genes on 135 of the 316 signaling pathways were DE between the 9311/*Xa21* and 9311 plants. These *Xa21*-mediated basal pathways included mainly those related to the basic material and energy metabolisms and many related to phytohormones such as cytokinin, suggesting that *Xa21* triggered redistribution of energy, phytohormones and resources among essential cellular activities before invasion. Counter-intuitively, after infection, the DE genes between the two plants were only one third of that before the infection; other than a few stress-related pathways, the affected pathways after infection constituted a small subset of the *Xa21*-mediated basal pathways. These results suggested that *Xa21* primed critically important genes and signaling pathways, enhancing its resistance against bacterial infection.

Rice (*Oryza sativa*) is one of the most widely consumed staple crops in the world, feeding about half of the world population. Rice bacterial blight (BB), caused by infection of *Xanthomonas oryzae* pv. *oryzae* (*Xoo*), is a devastating disease of rice, resulting in 20% to 30% annual reduction of rice production worldwide^1^,^2^. Chemical pesticides and biocontrol agents, such as plant extracts^3^ and chitosan solutions^4^, have been used to control BB. Besides the environmental and food safety issues that these biochemical agents can cause, the field effects of these agents are far from satisfactory and their effectiveness diminishes over time of usage. To date, the most effective and economic means to control BB disease is to introduce disease resistant genes into rice plants^1^.

A total of 22 dominant and 9 recessive BB resistant genes have been identified^2^,^5^, some of which have been widely used in rice production. Among these genes, *Xa21* is the most well studied. Through phosphorylation^6^,^7^ and cleavage of its intracellular kinase domain^8^, *Xa21*—a cell membrane receptor—perceives the presence of *Xoo* and relays the signal to the nucleus through multi-step signal cascades involving some key proteins such as XA21 Binding Protein 3 (XB3)^9^, mitogen-activated protein kinase 5 (MAPK5)^10^, MAPK12^10^, and transcription factors (TFs) including OsWRKY62 and OsWRKY76^10^,^11^,^12^ in the nucleus. Some *Xoo* resistant genes, such as *xa5*, are transcription factors^13^,^14^. Furthermore, many effectors from *Xoo* belong to the transcription activator-like (TAL) family, which facilitate injection into rice cells to activate susceptibility genes in the host to exert their functions^15^. The known *Xoo* effectors include *avrxa5*^16^, *avrXa7*^17^, *avrXa10*^18^ and *avrXa27*^19^, which trigger *xa5*-, *Xa7*-, *Xa10*- and *Xa27*-mediated resistance, respectively. Recently, *avrXa23* was cloned and shown to be a TAL effector^20^. Overall, this transcription-activating characteristic of the *Xoo* effectors suggested their role of disrupting gene transcription regulation after *Xoo* infection, resulting in reprograming of the transcriptome of the host, as observed by microarray gene profiling of resistant and susceptible genotypes^21^,^22^,^23^.

In light of the broad disease resistance spectrum and endurance as well as distinct metabolite profiles^24^, it is necessary to conduct an independent study that focuses primarily on the mechanisms of *Xa21*-conferred BB resistance. In this study, we exploited two isogenic rice genotypes, one with and the other without the *Xa21* gene, profiled their transcriptomes before and within the first 96 hours after *Xoo* infection, and contrasted the variations of the transcriptomes of the two rice lines in reference to the transcriptome of the normal rice plants. The analyses of the large quantities of gene expression profiling data from deep sequencing and a *de novo* genome sequencing result revealed the genes, biological processes and signaling pathways that are responsible for the resistance to rice blight infection.

## Results And Discussion

### A system for study of *Xa21* mediated BB resistance

To establish a platform for studying *Xa21*, we generated a nearly isogenic line (NIL) of rice *indica* variety 9311 that carries the desirable *Xa21* gene. In order to minimize or eliminate the possible impact of diverse genetic background on gene expression, we used the 9311 rice, which is susceptible to *Xoo*, as the recurrent parent and the CBB23 rice, which carries *Xa21*, as the donor, and successively applied more than 15 generations of backcrossing to introduce *Xa21* into 9311, creating a new line of 9311/*Xa21* rice. Fifteen generations of backcrossing are more than double the minimum of 6 backcrossing generations typically required to recover the phenotypes of the recurrent parental line and eliminate most of the donor chromosome fragments linked with the target *Xa21* gene. As a result, the 9311/Xa21 rice, referred to as the *R*(esistant) *plant*, should have nearly the same genetic background as the 9311 rice, denoted as the *S*(usceptible) *plant*, except that the former carried *Xa21* and its linked genomic fragment.

**Figure 1:**
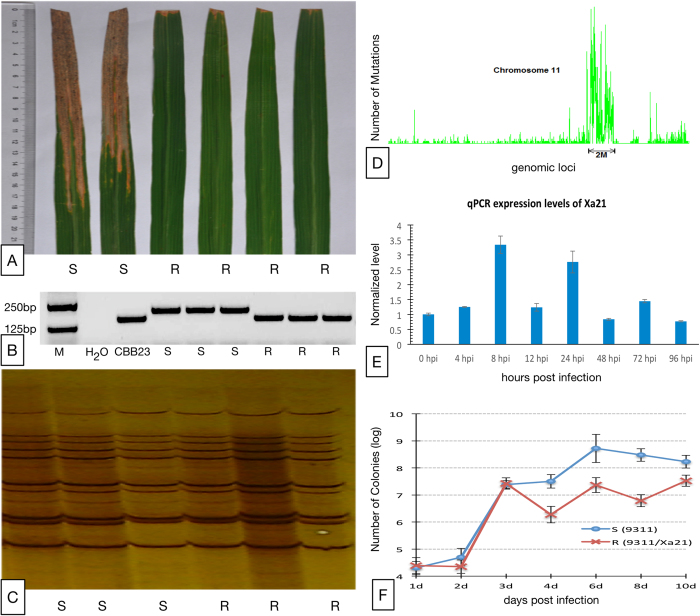
(**A**) Comparison of in-field phenotypes of the R and S lines after inoculation of the P6 strain of *Xoo*. (**B**) PCR validation of *Xa21* being introduced into the genome of the R (9311/Xa21) plants using the molecular marker U1/I2 that was co-segregated with *Xa21*. (**C**) The AFLP result of the R and S plants using 24 pairs of AFLP primers. (**D**) Genotypic variations across chromosome 11 of the R rice line. (**E**) The expression of *Xa21* in the R plants at various time points before and within the first four days after *Xoo* inoculation. (**F**) Comparison of the amount of bacteria, measured by the number of bacterial colonies, in the R and S plants in the first 10 days post inoculation of *Xoo*.

The nearly isogenic property between the R and S plants was further tested and validated using multiple means, including phenotyping, genotyping, whole genome sequencing and transcriptome profiling. The inclusion of *Xa21* in the R plants was first indicated by the substantially smaller lesions on the R plants than on the S plants after *Xoo* inoculation (Fig. 1A) and the presence of a molecular marker co-segregated with *Xa21* in the R plants (Fig. 1B). Furthermore, three lines of evidence showed the similar genetic backgrounds of the R and S plants except a small region encompassing the *Xa21* gene. The R and S plants shared many similar agronomic traits (Supplemental Table S1), exhibited similar AFLP (Amplified Fragment Length Polymorphism) profiles (Fig. 1C), and had nearly identical genome sequences, based on re-sequencing of their genomes (see Methods), on all chromosomes except a ~2 Mbp region encompassing *Xa21* on chromosome 11 of the R plants (Fig. 1D). In short, these results showed that the R and S rice lines were nearly isogenic.

The ~2 Mbp long *Xa21-hosting region,* which was inevitably introduced into the R plants along with *Xa21* through backcrossing, had a high rate of genotypic variation (Fig. 1D) and harbored a total of 250 genes, among which 90 had non-synonymous mutations. It is noteworthy that only one of the 90 non-synonymously mutated genes, except *Xa21*, was expressed. We suspect the functional impact of the genes in the *Xa21*-hosting region except *Xa21* is minimal. In support of this belief, we showed in a previous comparable study that a transgenic rice line and a rice line generated by backcrossing, both of which carry *Xa21*, were substantially equivalent at the transcriptome level^25^. Moreover, as validated by real time PCR, *Xa21* in the R plants was constitutively expressed across all of the eight time points before and after *Xoo* infection that we profiled (Fig. 1E). In contrast, the regulatory role of expressed *Xa21* on downstream genes was implicated by transcription factor *OsWRKY62*, which had a much lower abundance in the R plants than in the S plants before *Xoo* infection (Table S2). *OsWRKY62* directly interacts with the *Xa21* protein and acts as a negative regulator against *Xoo* in rice by suppressing defense-related genes^11^. Together, these results clearly showed that most of the genetic backgrounds of the R plant had been substituted with those of the S plants by backcrossing, and the R and S plants formed an ideal system for studying the functions of *Xa21*.

### *Xa21* suppressed *Xoo* growth

Our first step to characterizing the functions of *Xa21* was to profile the *in planta* growth of pathogen *Xoo* within both R and S plants in the first 10 days after pathogen inoculation (see Methods). No apparent difference of pathogen growth was observed between the R and S lines within the first 3 days while the pathogen was making its way into the host cells. However, starting on day 4 the growth of *Xoo* was substantially suppressed in the R plants with respect to the growth in the S plants (Fig. 1F). The suppression was statistically significant (t-test, p-value < 0.05) and persisted at least for 10 days after inoculation. This *in planta* observation revealed *Xa21* as a suppressor of *Xoo* growth so as to curtail its virulence.

### *Xoo* triggered broad perturbation in rice transcriptomes

In order to investigate how *Xa21* responded to *Xoo* infection, we adopted high-throughput deep-sequencing to profile the transcriptomes of both R and S lines before *Xoo* infection and at seven time points within the first 4 days after pathogen inoculation (see Methods). Sequencing profiling produced more than 594 million raw reads from 19 plant samples (Table S3). About 82.24% (±1.29%) of the raw reads can be mapped to the rice reference genome and more than 92.10% (±1.26%) of the qualified reads (after removing low-mapping-quality reads) can be mapped to the exon regions of annotated genes (Table S3). This sequencing-based profiling provided a deep and broad map of transcriptome variations of the R and S rice plants in the process of *Xoo* infection.

**Figure 2:**
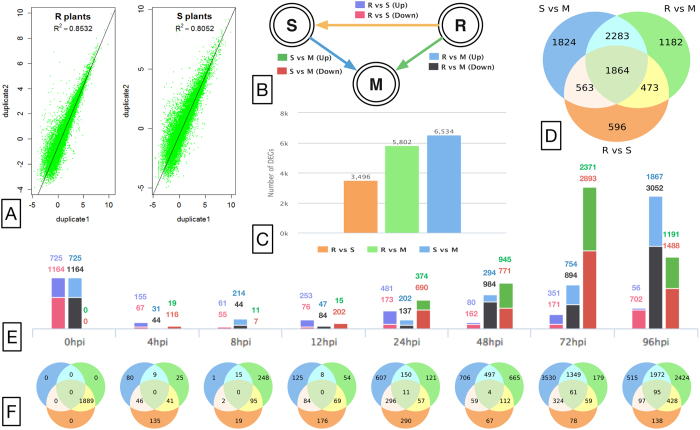
(**A**) Correlations between the duplicates of the R plants (left) and of the S plants (right). (**B**) The scheme for differential expression analysis. A circle represents a type of three libraries, i.e. *Xoo* susceptible 9311 (S), *Xoo* resistant 9311/Xa21 (R), and mock control 9311 (M). An edge represents a DE comparison, i.e., R vs S, S vs M, and R vs M. Different types of comparison are shown in different colors. (**C**) The number of unique DE genes in each DE comparison. (**D**) The numbers of overlapped DE genes among the comparisons. (**E**) The number of DE genes at each time point. (**F**) The numbers of overlapped DE genes among the comparisons at each time point.

Gene expression profiles of biological duplicates of the R and S plants before *Xoo* inoculation were produced to assess the quality of deep-sequencing based gene expression profiling. The result showed that the expression profiles were highly reproducible, with the Pearson correlation coefficients between the duplicates of the R plants and between the duplicates of the S plants being 0.8532 and 0.8052, respectively (Fig. 2A).

We analyzed gene expression levels to characterize transcriptome variations in the period of the first four days of infection (see Methods) between the R plants inoculated with *Xoo* and the 9311 rice (the S line) grown under the normal condition—the Mock—and between the S plants infected with *Xoo* and the Mock (Fig. 2B). Particularly, 5,802 and 6,534 differentially expressed (DE) genes existed for at least one time point in the first 4 days after pathogen inoculation in the R and S plants with respect to the Mock, respectively (Fig. 2C), indicating that Xoo infection triggered, in genome scales, transcriptomic perturbations in these two plants. All of these DE genes are listed in a file available at http://www.cse.wustl.edu/~zhang/software/xa21DEgenes.zip.

Remarkably, the two transcriptomic perturbations were incompatible, even if the difference between the two rice lines was only due to the ~2 Mbp *Xa21*-hosting region in the R plants. A total of 3,496 genes exhibited significant expression variations between the R and S plants for at least one of the seven time points after *Xoo* infection (Fig. 2C,D and Table 1), and most of these DE genes were aggregated into 187 of the 316 (59.18%) annotated pathways in rice (RiceCyc pathway database, version 3.3, Dharmawardhana, Ren *et al.* 2013) (Table S4); these 187 DE-gene containing pathways were referred to as the *Xa21-induced pathways*. These results of distinct transcriptomic responses suggested that *Xoo* activated different signaling pathways in the two rice plants, resulting in their distinct *Xoo* resistances (Fig. 1A,F).

**Figure 3:**
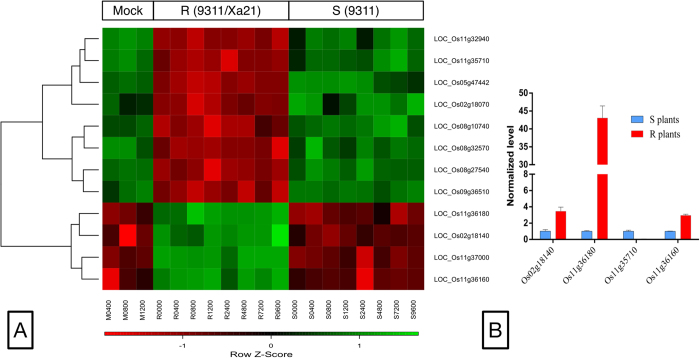
(**A**) The heatmap of the expression patterns of 12 rice genes, which were constantly up- or down-regulated throughout the infection of *Xoo*. (**B**) Real-time quantitative PCR validations of four genes continuously up- or down-regulated across the R and S plants.

Since the R rice exhibits stable resistance to *Xoo* throughout all stages of rice development, it is viable to hypothesize that genes continuously up-regulated or down-regulated across the R and S plants were most likely to be related to the resistance of *Xa21*. Along the seven time points profiled during the infection of *Xoo*, 12 genes were consistently differentially expressed between the R and S plants, where 4 genes were highly induced and the remaining 8 suppressed in the R plants (Fig. 3A). Four of these 12 genes (LOC_Os02g18140, LOC_Os11g36180, LOC_Os11g35710, LOC_Os11g36160) were further examined by real-time PCR for their expression variations across the R and S plants before *Xoo* inoculation (Fig. 3B). Note that LOC_Os02g18140 encodes a NBS type disease resistance protein and the other three genes are on chromosome 11 where *Xa21* resides. LOC_Os11g36160 and LOC_Os11g36180 were particularly interesting since they reside in the neighborhood of *Xa21*, were highly induced in the R plants, and were annotated to be receptor kinases.

### *Xa21* mediated complex basal signaling pathways to prepare for *Xoo* infection

Besides the broad, distinct perturbations to the transcriptomes of the R and S rice caused by *Xoo* infection, the most surprising result of the transcriptome profiling was a great deal of transcriptomic difference between the R and S rice *before bacterial infection*. Precisely, 1,889 genes, involving in 135 signaling pathways, exhibited significant expression variations between the R and S plants before *Xoo* inoculation (Fig. 2E). This is remarkable as it clearly indicated that *Xa21* was already functional before the infection. These 135 pathways, referred to as the *Xa21-mediated basal pathways*, were related to various types of material and energy matabolisms (Table S4). Among them, 28, 26 and 4 *Xa21*-mediated basal pathways were related to basic material and energy metabolisms, cellular components, and synthesis metabolisms, respectively. In contrast, based on Gene Ontology, many *Xa21*-induced processes after infection were directly related to stress responses and infection (Table 2), including all kinds of phytohormones and phytoalexins, whereas the first stress related biological process was only ranked the 24th among the *Xa21*-mediated basal processes (Table S5).

It is worthwhile to mention that at any of the seven time points after *Xoo* infection that we profiled, the degrees of differential expression between the R and S plants were substantially less than before the infection (Fig. 2E, the first columns of all time points). For example, at 8 hours post inoculation (hpi), 116 genes were differentially expressed, which were only 6.25% of the 1,889 DE genes before the infection; these 116 DE genes were involved in 10 signaling pathways (Table 3), in sharp contrast to the 135 *Xa21*-mediated basal pathways. On the other hand, approximately 66.6% of the *Xa21*-mediated basal pathways overlapped with the *Xa21*-induced pathways (Table S4). This high degree of overlap suggested that, even before *Xoo* infection, *Xa21* had prepared the R rice well so that it was able to respond to the presence of *Xoo* as effectively and quickly as possible, as illustrated by its strong resistance to *Xoo* (Fig. 1).

Together, these results suggested that before *Xoo* invasion*, Xa21* in the R rice effectively reallocated energy and resources among many house-keeping cellular activities to prepare the plant, and after infection, *Xa21* adjusted or activated stress related pathways according to a given *Xoo* strain and the time of pathogenesis.

### Cytokinins contribute to *Xa21* resistance to *Xoo*

The most notable enriched pathways between the R and S plants at various time points after *Xoo* inoculation were the ones related to phytohormones (Table 4 and S4). The pathways related to jasmonic acid (JA) and ethylene (ET), two classic phytohormones, had many DE genes in two of the eight time points profilied. Meantime, the pathways of some other members of hormone families, such as brassinosteroids (BR), gibberellic acid (GA) and cytokinins (CK), were also enriched (Table 4). Some phytohormones regulated themselves through different pathways at different time points. For example, at 4 hpi, the GA biosynthesis pathway had a substantial number of DE genes, however, at 24 hpi, the pathway for gibberellin inactivation was enriched. Furthermore, different DE genes were involved in the same pathways of the same hormones and showed distinct expression patterns in the R and S plants at different time points in the first four days of infection (Table S4).

**Figure 4:**
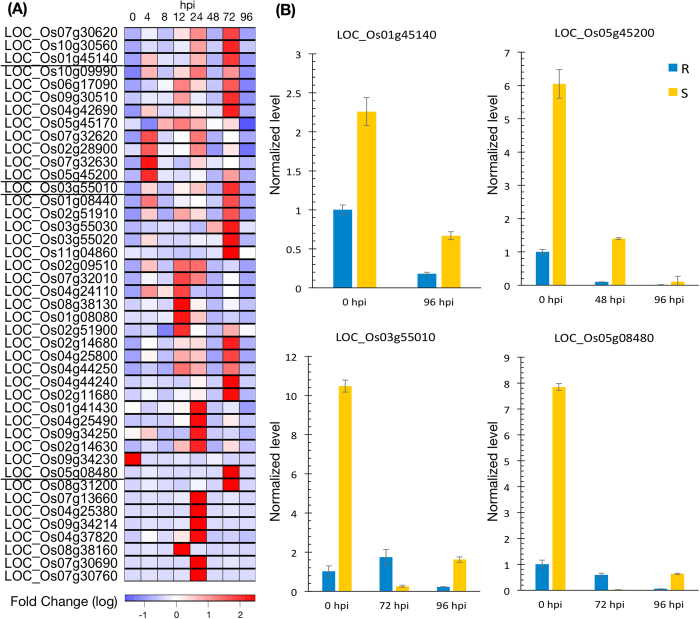
(**A**) The relative fold-change of cytokinins genes that were significantly differentially expressed for at least one time point. Red and blue corresponds to down-regulated or up-regulated fold-change, respectively, for each gene in the first 96 hours post infection. (**B**) Real-time quantitative PCR validated the expression levels of four genes in the cytokinins-related pathways, which were identified as significantly DE at a given time point.

Among the phytohormones detected by the transcriptome profiling, CK was the most pronounced. The DE genes residing on the CK pathways appeared at five of the seven time points after *Xoo* infection, four of which were the highest ranked among all pathways detected (Table S4), alluding to CK’s involvement in *Xa21*-mediated resistance to *Xoo*. These CK pathways included that for cytokinins-O-glucoside biosynthesis (PWY-2902), cytokinins-9-N-glucoside biosynthesis (PWY-2901), and cytokinins-7-N-glucoside biosynthesis (PWY-2881), which were responsible for CK conjugation and thus for inactivating cytokinins^26^. CK is known to be involved in plant disease resistance^27^ and in biotic^28^ and abiotic^29^,^30^,^31^ stress responses. The DE genes on the cytokinins-glucoside biosynthesis pathways exhibited a relatively coherent pattern of expression (Fig. 4A). Before *Xoo* infection, most of the DE genes, i.e., 15 of the 16 (93.75%), had lower abundances in the R plants than in the S plants. In contrast, at the 12, 24 and 72 hpi, 26 of the 27 (96.30%) DE genes on the cytokinins-glucoside biosynthesis pathways had higher abundances in the R plants than in the S plants. At 96 hpi, all (100%) of the 18 DE genes on the cytokinins-glucoside biosynthesis pathway had lower abundance in the R plants. For example, the low expression abundances of 4 genes in the CK-related pathways were validated by real time PCR (Fig. 4B). Such a high degree of coherent expression pattern over the course of *Xoo* infection suggested that the role of endogenous CK in *Xa21-*mediated resistance is complex and is difficult to be imitated by a constant amount of exogenous CK or by constantly inducing/repressing CK-related genes, which was often adopted in the previous studies on the functions of endogenous phytohormones.

### Pterocarpan phytoalexins might function to repress *Xoo* in the R plants

**Figure 5:**
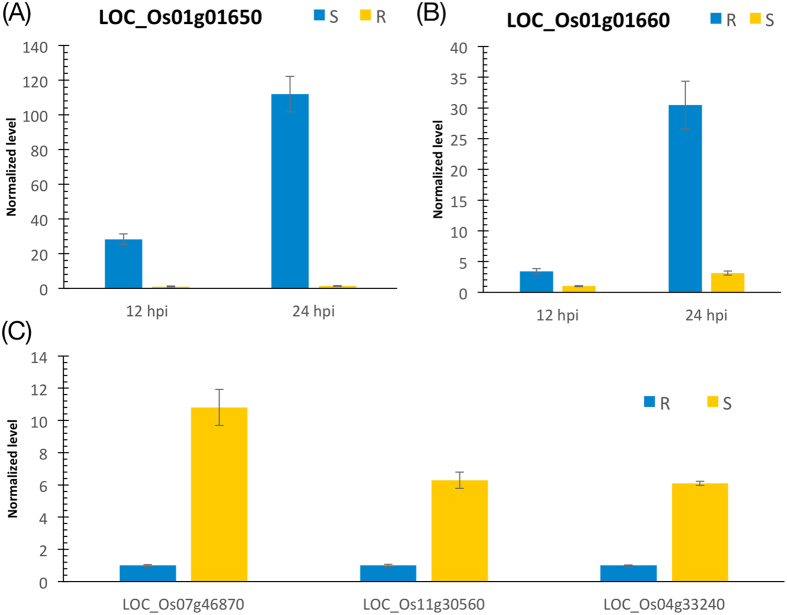
(**A**,**B**) Real-time quantitative PCR validated two genes in the pathway of pterocarpan phytoalexin. LOC_Os01g01650 and LOC_Os01g01660 were significantly induced in the R plants at 12 and 24 hpi. (**C**) Real-time quantitative PCR validated three genes in enterobactin biosynthesis pathway. LOC_Os07g46870, LOC_Os11g30560, and LOC_Os04g33240 were significantly repressed in the R plants without the infection.

Maackiain, together with medicarpin, is the main pterocarpan phytoalexin in chickpea and occurs exclusively in a (-)-(6aR,11aR)-configuration^32^. Two adjacent genes on rice chromosome 1, LOC_Os01g01650 and LOC_Os01g01660, encode the enzymes for production of medicarpin and maackiain, respectively. These two genes had similar expression patterns in the R and S plants before *Xoo* infection. At the initial *Xoo* infection, their expressions were dramatically repressed at 4 hpi in both R and S plants. The suppression of these two genes persisted in the S plants throughout the 96 hours of infection. These results suggested that *Xoo* suppressed the synthesis of pterocarpan phytoalexins to promote its growth and disease progression in the S plants. However, the expressions of these two genes in the R plants elevated dramatically after 12-hpi and peaked at 24-hpi, which were further validated by real time PCR (Fig. 5A,B). The expression levels were suppressed again after 24-hpi and dropped to nearly 0 at 96-hpi. The increased expression of the two genes in the R plants might promote the synthesis of pterocarpan phytoalexin to control BB disease within the early stage of 24-hpi.

Notably, these two genes were not highly expressed in a rice line that is susceptible to rice blast fungal infection after the fungal infection (data not shown), suggesting that pterocarpan phytoalexins responded differently to fungal and bacterial infections. In addition, mock infection with water did not induce these two genes in the S plants either, confirming that they responded to *Xoo* infection.

### Involvement of iron in *Xa21*-mediated disease responses

Iron is a key nutrient for bacterial growth, and the usable form of iron for microorganisms is usually siderophore^33^. As *Xoo* colonizes within rice xylem, where siderophores are derived, the host typically exploits the essentiality and toxicity of transition metals to defend against bacterial invaders^34^. Before *Xoo* infection, the pathway of the enterobactin biosynthesis, a catecholate siderophore, was significantly enriched with 12 DE genes (FDR = 0.08703, Table S4), among which, 11 genes had lower abundance in the R plants. The experimental validation of three of the DE genes, using real-time quantitative PCR was consistent with the result from sequence profiling (Fig. 5C). The reduced expressions of these genes on the enterobactin biosynthesis pathway might help lower the amount of siderophores, which in turn restricted *Xoo* colonization in the R plants after *Xoo* infection. Because of iron uptake deficiency, a mutation in the *Xoo feoB* gene causes severe virulence deficiency and constitutive production of a siderophore^35^. A defect in siderophores formation in *Dickeya dadantii*, a plant soft-rotting enterobacterium, leads to symptoms localized to inoculated leaves, indicating that the siderophores are required for bacteria to spread to the other parts of the plant^36^,^37^. The R plants had limited blight lesions on inoculated leaves (Fig. 1), indicating that *Xoo* was curtailed in the R plants. Since most known mechanisms of disease resistance through iron-withholding are realized by regulation of iron-binding proteins^33^,^38^,^39^, it is viable to hypothesize that siderophores were also restricted in the R plants through iron-withholding. The way to restrict active iron in the form of siderophore seemed to be rare; this may be a double-edged sword because the lack of active iron is harmful to *Xoo* as well as to the rice plant at the same time.

### Concluding remarks

The 9311 rice (the S line) and its nearly isogenic line with the *Xa21* gene (the R line) that we used form a robust tool for studying the function of *Xa21*. In combination of deep sequencing-based transcriptome profiling and bioinformatics analysis, our results provided remarkable genome-wide profiles of gene expression and related signaling pathways and biological processes that significantly differed in the two rice genotypes. The significant difference between the transcriptomes of the two rice genotypes before *Xoo* infection revealed insights into the functions of *Xa21* in priming various metabolic pathways so as to gain high and durable resistance to *Xoo*. After *Xoo* infection, *Xa21* mediated DE genes and pathways were sharply reduced but more related to resistance to *Xoo*. Among them, the plant hormones, especially cytokinins, were broadly involved, suggesting complex mechanisms of hormones in *Xa21*-mediated resistance to *Xoo*.

## Material and Methods

### Rice varieties and growth condition

Rice 9311 variety, a popular *indica* rice restorer line^40^, and the backcrossing line 9311/*Xa21* were used to study *Xa21*-mediated BB resistance. The 9311 rice was susceptible (the S genotype) and the 9311/Xa21 rice resistant (the R genotype) to *Xoo* infection. These two rice lines have identical genetic background except the latter carrying *Xa21*. Seeds of the two plants with no defects nor disease were surface-sterilized in 70% ethanol for 2 min, rinsed twice in deionized water, imbibed overnight at 30 °C and placed on moist filter paper overnight at 30 °C. Germinated seeds were then sown in UC potting mix. Pots with plants were kept in nutrient solution (0.25 × Hoagland’s solution) in a greenhouse in Hainan province (lat 20° 1’ N, long 110°19’ E). The greenhouse conditions in which the rice seedlings were cultivated were: ~30 °C/20 °C (day/night), ~80% RH, natural sunlight with a ~13 h/11 h light/dark photoperiod at ~40 W/m^2^ intensity.

### Pathogen inoculation and evaluation

*Xanthomonas oryzae* pv. *oryzae* (*Xoo*) Philippine race 6 (P6) was used for pathogen inoculation. *Xoo* was subcultured at 28 °C on PSA (Potato-Sugar-Agar) medium (potato, 300 g/L; Ca(NO_3_)_2_•4H_2_O, 0.5 g/L; Na_2_HPO_4_•12H_2_O, 2.0 g/L; sugar, 15 g/L; agar, 15 g/L) for 3 days. Inoculums were prepared by suspending the bacterial cells in sterile water and adjusting the concentration to about 10^9^ cells per milliliter. The last rice leaves were infected with P6 by using scissors dipped in bacterial suspensions to clip leaves 1–2 cm down from the tip of the leaf blade at the heading stage of 9311 and 9311/*Xa21*^41^. Mock-infected plants were treated in a similar fashion except that water substituted for P6. Fifteen days post inoculation, lesion length was measured from the cut surface at the tip to the distal-most position on the leaf that exhibited a grey, chlorotic or water-soaked lesion.

### DNA and RNA isolation and genetic analysis

Fifteen randomly selected leaves were harvested at each time point (Table 1) and pooled to represent each treatment. After harvest, leaves were immediately frozen and stored in liquid nitrogen until use. About 100 mg samples were grinded to powder with liquid nitrogen for DNA and total RNA isolation using the Total DNA/RNA Isolation Kit (R6731, Omega, USA) following the manufacture’s protocols. The total RNA quality was measured using Agilent RNA 6000 Pico Kit (5067–1513, Agilent, USA). Only the total RNAs with RIN (RNA Integrity Number) no less than 7 were used for the subsequent experiments.

To investigate whether *Xa21* was introduced into the genome of the R plant, we designed a molecular marker (Sequence listed in Table S6), named as U1/I2, which was co-segregated with *Xa21*. This marker was able to amplify a fragment of 575 bp in the R plant and a fragment of 445 bp in the S plant.

The method for analyzing the expression of *Xa21* was described in our early report^42^; Amplified fragment length polymorphism (AFLP) was used for genetic background analysis for the R and S genotypes, using the methods of Vos *et al.*^43^. AFLP primers were given in our earlier report^44^.

### Assay for quantification of bacterial growth

The bacterium population was determined using three P6-infected leaf samples collected at 0d (day), 1d, 2d, 3d, 4d, 6d, 8d and 10d after inoculation. The bacterial growth was analyzed according to Song *et al.*^45^ with three biological replicates.

### DNA and RNA library preparation, emulsion PCR and sequencing

About 1 μg genomic DNA from the R and S plants were used for DNA library preparation, using the SOLiD™ 5500 Fragment Library Core Kit (Part no. 4464412) according to its user guide. A total of 20 μg total RNA was used for two rounds of mRNA purification using Dynabeads (610.06, Invitrogen, USA). About 100 ng mRNA was fragmented using NEB Next Magnesium RNA Fragmentation Module (E6150, NEB), purified with an RNA clean up kit (R6247, Omega, USA), end repaired with T4 Polynucleotide Kinase (T4 PNK) (M0201, NEB) and cleaned up again with a kit (R6247, Omega). The end-repaired RNAs were used to prepare the strand specific transcriptome, using the Small RNA Sample Preparation kit (E6160, NEB) according to the manufacturer protocol with some minor modification, including the SR Primer F3 being replaced with barcode primers. The resulting DNA and RNA libraries were used for emulsion PCR to produce the beads for sequencing on the SOLiD 5500 machine, using 75 nt mode and 75 nt + 35 nt mode for the sequencing of DNA and RNA libraries, respectively. Biological duplicates of RNA libraries of the R and S plants before *Xoo* infections were profiled for quality assessment.

### Analysis of genotypic variation

The DNA sequencing reads in the color-space format were mapped to the Oryza sativa *Nipponbare* reference genome and gene annotation from MSU Rice Genome Annotation Project (Release 7^46^) using LifeScope (Life Technologies) software version 2.5.1.

Genotypic variations in the R rice line were analyzed using the genomic regions where the genome sequences of the S line and the 9311 reference genome were the same in order to rule out possible impact of natural mutations in the S plants. The analysis had a resolution of 5 Kbp, in which sequence variations within a 5 Kbp window were tallied. The calling of a genotypic variation at a genomic locus of the R line was subjected to a set of stringent criteria: the locus had distinct nucleotides in the R and S lines; and the sequencing must have at least 5X coverage of the same read at the locus to rule out or minimize possible sequencing error.

### Gene expression and differential expression analysis

For RNA analysis, the reads mapped to each annotated gene were tallied using the whole transcriptome analysis workflow of LifeScope. Differential expression analysis was performed using the edgeR^47^ package. The reads count per gene was normalized using the TMM method in edgeR. An exact test, analogous to the Fisher’s exact test, was performed based on the normalized counts with the common dispersion factor being set to 0.1. Genes were considered to be differentially expressed if they had more than 10 read counts across all samples and their False Discovery Rates (FDR)^48^ of the exact test were no greater than 10%.

### Functional and pathway enrichment analysis

The Gene Ontology^49^ Slim annotations of rice genes were downloaded from the MSU Rice Genome Annotation Project^46^. The association information of rice genes and pathways was retrieved from the RiceCyc pathway database (version 3.3, Dharmawardhana, Ren *et al.* 2013). Given a list of genes, the Fisher’s exact test was performed to measure the statistical significance of enrichment of the genes on a GO term or pathway; the resulting *p* value was subject to Benjamini-Hochberg multiple testing correction^50^ to derive the final FDR.

### Gene expression validation

Real-time PCR was performed following the standard THUNDERBIRD SYBR qPCR Mix kit (QPS-201, Toyobo) protocol. The 20-μl reactions (0.3-μl first-stand cDNA product, 10-μl THUNDERBIRD qPCR Mix, 0.3 μM forward and reverse primers, 1 × ROX reference dye) were incubated in 0.2 ml tubes of Applied Biosystems StepOne™ Real-Time PCR machine as follows: 95 °C for 20 s, followed by 40 cycles of 95 °C for 5 s and 60 °C for 30 s. The procedure ended by a melt-curve ramping from 60 to 95 °C, raised by 0.5 °C each step. The primers used for real time PCR are listed in Table S6. Data were normalized using the reference gene LOC_Os06g11170.1 (coding for a putative nucleic acid binding protein) with the same primers published by Narsai *et al.*^51^. Before performing expression analysis, the primers’ efficiency was estimated through a five-point standard curve. Only the target genes with amplification efficiencies between 90%–105% were chosen for expression validation. All PCR reactions were done in three biological replicas and three technical replicas. Ct values were exported and analyzed using Microsoft Excel 2010.

## Additional Information

**How to cite this article**: Peng, H. *et al.* Rice *Xa21* primed genes and pathways that are critical for combating bacterial blight infection. *Sci. Rep.* **5**, 12165; doi: 10.1038/srep12165 (2015).

## Supplementary Material

Supplementary Information

## Figures and Tables

**Table 1:** Plant samples and hours post inoculation (hpi) when transcriptomes were profiled.

hpi	9311	9311/Xa21	Mock(9311)
0 h	S0	R0	
4 h	S4	R4	M4
8 h	S8	R8	M8
12 h	S12	R12	M12
24 h	S24	R24	
48 h	S48	R48	
72 h	S72	R72	
96 h	S96	R96	

**Table 2:** The numbers of significant GO Terms that were related to infection response at different time points. Rankings were based on the FDR values of enrichment test.

Term Name	0 h	4 h	8 h	12 h	24 h	48 h	72 h	96 h
response to stimulus	43	3	1	20	20	1	2	4
response to stress	40	4	2	15	18	7	6	17
response to biotic stimulus	39	15	20	23	24	55	35	5
response to abiotic stimulus	24	16	40	48	31	56	3	15

**Table 3:** Overlap between the *Xa21*-mediated basal pathways and the perturbed pathways after *Xoo* infection.

Time (hpi)	0 h	4 h	8 h	12 h	24 h	48 h	72 h	96 h
Overlapped pathways	135 (100.00%)	22 (70.97%)	7 (70.00%)	18 (66.67%)	54 (72.00%)	34 (82.93%)	54 (69.23%)	51 (79.69%)
All perturbed pathways	135	31	10	27	75	41	78	64

**Table 4:** Enriched hormone pathways (p-value < 0.05) related to the *Xa21*-mediated *Xoo* responses.

Time	0 h	4 h	8 h	12 h	24 h	48 h	72 h	96 h
cytokinin	√			√	√		√	√
ethylene	√		√					
gibberellin		√			√			
jasmonic acid		√						√
IAA					√			√
brassinosteroid						√		
