# Supplementary material for: Rice Xa21 primed genes and pathways that are critical for combating bacterial blight infection
Source: Sci Rep. 2015 Jul 17;5:12165. doi: 10.1038/srep12165 (PMC4505318; doi:10.1038/srep12165)
Supplement: Supplementary Information [file srep12165-s1.pdf]

Rice *Xa21* primed genes and pathways that are critical for combating bacterial blight infection – Supplemental Tables

Hai Peng[1*], Zheng Chen[1,2*], Zhiwei Fang[1], Junfei Zhou[1], Zhihui Xia[3], Lifen Gao[1], Lihong Chen[1], Lili Li[1], Tiantian Li[1], Wenxue Zhai[4] and Weixiong Zhang[1,2,5†]

[1] Institute for Systems Biology, Jianghan University, Wuhan, Hubei 430056, China;

[2] Department of Computer Science and Engineering, Washington University in St. Louis, St. Louis, MO 63130, USA;

[3] Hainan Key Laboratory for Sustainable Utilization of Tropical Bioresources, Institute of BioScience and Technology, College of Agriculture, Hainan University, Haikou 570228, China;

[4] Institute of Genetics and Developmental Biology, Chinese Academy of Science, Beijing 100101, China;

[5] Department of Genetics, Washington University in St. Louis, St. Louis, MO, 63130, USA.

* Equal contribution.

† Correspondence – weixiong.zhang@wustl.edu.

Table S1

Major agronomic traits between R and S genotype

Genotype	Lesion Length(cm)	Plant height(cm)	Panicles per plant	Panicle length(cm)	Filled grains per panicle	Seed ratio (%)	1000-grains weight(g)
S	15.22±1.40	110.07±7.67	6.21±2.02	21.02±3.06	128.41±22.45	82.21±4.23	32.01±0.62
R	0.54±0.23**	112.07±8.02	6.56±3.33	21.56±2.89	135.66±18.74	80.96±5.61	32.36±0.52

** represents a significant level of $p < 0.01$ between R and S genotype

Table S2. The expression of *OsWRKY62* in the R and S plants before *Xoo* infection

Plant type	Duplicate 1	Duplicate 2	Average	log2 (R/S)
R plants	0.28	0.33	0.31	-2.40
S plants	1.45	1.77	1.61	

Table S3. RNA-Seq Alignment Summary

Lib	Total Reads	Unmapped Read	Unmapped Read %	Mapped Read %	Low quality Reads (MapQV<10)	Qualified read	Qualified read %	Exon Reads	Exon Reads in Qualifed read %	Intron Reads	Intergenic Reads
M0400	13,657,244	2,225,889	16.30%	83.70%	3,145,462.00	8,285,893	60.67%	7,532,837	90.91%	268,312	484,744
M0800	23,534,874	4,050,582	17.21%	82.79%	4,006,581.00	15,477,711	65.77%	14,166,535	91.53%	575,417	735,759
M1200	23,582,358	4,201,732	17.82%	82.18%	4,261,563.00	15,119,063	64.11%	13,925,291	92.10%	469,627	724,145
R0000	31,109,398	5,846,116	18.79%	81.21%	14,817,322.00	10,445,960	33.58%	9,490,032	90.85%	249,227	706,701
R0400	31,167,940	5,836,735	18.73%	81.27%	4,920,670.00	20,410,535	65.49%	18,893,296	92.57%	523,725	993,514
R0800	93,404,954	18,911,008	20.25%	79.75%	10,197,048.00	64,296,898	68.84%	59,348,453	92.30%	2,140,663	2,807,782
R1200	20,837,024	3,746,240	17.98%	82.02%	2,434,934.00	14,655,850	70.34%	13,660,831	93.21%	375,633	619,386
R2400	51,069,596	9,142,468	17.90%	82.10%	5,235,689.00	36,691,439	71.85%	34,364,436	93.66%	641,079	1,685,924
R4800	20,124,746	3,208,945	15.95%	84.05%	4,307,073.00	12,608,728	62.65%	11,610,898	92.09%	309,884	687,946
R7200	32,202,048	5,901,692	18.33%	81.67%	4,905,074.00	21,395,282	66.44%	19,731,520	92.22%	588,588	1,075,174
R9600	15,609,474	2,480,854	15.89%	84.11%	3,407,208.00	9,721,412	62.28%	8,690,179	89.39%	439,629	591,604
S0000	21,440,282	3,933,074	18.34%	81.66%	4,581,755.00	12,925,453	60.29%	11,939,605	92.37%	248,977	736,871
S0400	13,899,090	2,487,160	17.89%	82.11%	4,601,688.00	6,810,242	49.00%	6,026,860	88.50%	231,674	551,708
S0800	85,458,550	17,714,996	20.73%	79.27%	9,017,929.00	58,725,625	68.72%	54,548,915	92.89%	1,610,344	2,566,366
S1200	15,289,962	2,635,506	17.24%	82.76%	3,430,844.00	9,223,612	60.32%	8,351,773	90.55%	347,700	524,139
S2400	8,768,102	1,430,772	16.32%	83.68%	2,320,648.00	5,016,682	57.22%	4,539,916	90.50%	132,612	344,154
S4800	30,447,808	5,532,732	18.17%	81.83%	5,153,984.00	19,761,092	64.90%	18,207,816	92.14%	492,266	1,061,010
S7200	15,811,724	2,580,124	16.32%	83.68%	2,690,769.00	10,540,831	66.66%	9,624,834	91.31%	390,561	525,436
S9600	46,696,134	8,070,810	17.28%	82.72%	10,431,196.00	28,194,128	60.38%	25,603,325	90.81%	970,628	1,620,175
Average	31,269,016	5,786,181	17.76%	82.24%	5,466,707	20,016,128	62.08%	18,434,597	92.10%	579,292	1,002,239
Std	22,635,796	4,726,997	1.29%	1.29%	3,202,485	16,100,906	8.43%	14,965,689	1.26%	488,749	674,516

Table S4 (A). RiceCyc Pathways of DE rice genes in Basal (0 hour)

Pathway ID	Pathway Name	Num	Expected Num	p-val	FDR
FERMENTATION-PWY	mixed acid fermentation	15	82	0.000118642	0.0160167
PWY-5082	methionine degradation III	11	55	0.000446845	0.0207199
P127-PWY	ethanol fermentation to acetate	12	64	0.000460443	0.0207199
PWY66-21	oxidative ethanol degradation I	11	57	0.00061725	0.0208322
PWY-5079	phenylalanine degradation III	11	61	0.001122579	0.0303096
TCA	TCA cycle	7	33	0.003550876	0.0684812
PWY-5188	tetrapyrrole biosynthesis I	7	33	0.003550876	0.0684812
TRESYN-PWY	trehalose biosynthesis I	5	19	0.005137846	0.0867012
ENTBACSYN-PWY	enterobactin biosynthesis	12	85	0.005801989	0.0870298
P23-PWY	reductive TCA cycle I	6	28	0.006539945	0.0882893
P105-PWY	TCA cycle variation I	7	39	0.009303139	0.1141749
HEME-BIOSYNTHESIS-II	heme biosynthesis II	4	15	0.011742445	0.1321025
LEUSYN-PWY	leucine biosynthesis	4	17	0.018561039	0.1814659
PWY-5473	hydroxycinnamic acid serotonin amides bio	3	10	0.020871736	0.1814659
DENOVOPURINE2-PWY	purine nucleotides <i>de novo</i> biosynth	8	56	0.021472152	0.1814659
THISYN-PWY	thiamine biosynthesis	2	4	0.021507064	0.1814659
ETHYL-PWY	ethylene biosynthesis from methionine	4	19	0.027415903	0.2177145
THRDLCTCAT-PWY	threonine degradation III (to methylglyoxal)	3	12	0.034889791	0.2355061
THREONINE-DEG2-PWY	threonine degradation II	3	12	0.034889791	0.2355061
PWY-5448	aminopropanol biosynthesis	3	12	0.034889791	0.2355061
PWY-2902	cytokinins-<i>O</i>-glucoside biosynthesis	16	162	0.042938549	0.2520306
PWY-2901	cytokinins 9-<i>N</i>-glucoside biosynthesi	16	162	0.042938549	0.2520306
PWY-2881	cytokinins 7-<i>N</i>-glucoside biosynthesi	16	162	0.042938549	0.2520306
PYRIDOXSYN-PWY	pyridoxal 5'-phosphate biosynthesis	2	6	0.049474996	0.2782969
ILEUDEG-PWY	isoleucine degradation I	3	14	0.052668137	0.2844079
PWY0-1182	trehalose degradation II (high osmolarity)	1	1	0.062615784	0.3200657
PWY-581	IAA biosynthesis I	5	35	0.064013144	0.3200657
PWY-841	purine nucleotides <i>de novo</i> biosynth	5	39	0.093136511	0.449051
PWY0-166	de novo biosynthesis of pyrimidine deoxyrib	3	19	0.111797398	0.5204362
PWY-2582	brassinosteroid biosynthesis II	9	92	0.118371429	0.5291755
PWY-361	phenylpropanoid biosynthesis	4	31	0.125132348	0.5291755
PWY66-162	oxidative ethanol degradation III	2	10	0.126015152	0.5291755
CHLOROPHYLL-SYN	chlorophyllide <i>a</i> biosynthesis	4	32	0.13642076	0.5291755
LEU-DEG2-PWY	leucine degradation I	2	11	0.147929262	0.5291755
GLYCLEAV-PWY	glycine cleavage complex	2	11	0.147929262	0.5291755
NONMEVIPP-PWY	methylerythritol phosphate pathway	2	12	0.170537156	0.5291755
PWY-2261	ascorbate glutathione cycle	2	12	0.170537156	0.5291755
PWY-3921	pantothenate biosynthesis II	2	12	0.170537156	0.5291755
PANTO-PWY	pantothenate biosynthesis I	2	12	0.170537156	0.5291755
PWY-5043	salvage pathways of purine nucleosides II (p	3	23	0.170992429	0.5291755
PWY0-181	salvage pathways of pyrimidine deoxyribon	1	3	0.176391834	0.5291755
NAGLIPASYN-PWY	lipid-A-precursor biosynthesis	1	3	0.176391834	0.5291755
THIOREDOX-PWY	thioredoxin pathway	1	3	0.176391834	0.5291755
PWY-1141	xanthophyll cycle	1	3	0.176391834	0.5291755
PWY-5059	pinobanksin biosynthesis	1	3	0.176391834	0.5291755
VALSYN-PWY	valine biosynthesis	3	24	0.186991296	0.5487788
ILEUSYN-PWY	isoleucine biosynthesis from threonine	3	25	0.203361522	0.5496913
MALATE-ASPARTATE-SHUTTLE-P	aspartate degradation II	2	14	0.217167361	0.5496913
DETOX1-PWY	removal of superoxide radicals	2	14	0.217167361	0.5496913
PWY-181	photorespiration	3	26	0.220050624	0.5496913
PWY0-162	de novo biosynthesis of pyrimidine ribonucl	3	26	0.220050624	0.5496913
PWY-40	putrescine biosynthesis I	1	4	0.228020091	0.5496913
DRIBOPMET-PWY	(deoxy)ribose phosphate degradation	1	4	0.228020091	0.5496913
PWY0-823	arginine degradation III (arginine decarboxy	1	4	0.228020091	0.5496913
GLUCUROCAT-PWY	β-D-glucuronide degradation	1	4	0.228020091	0.5496913
PWY-5034	GA₁₂ biosynthesis	1	4	0.228020091	0.5496913
PWY-5290	secologanin and strictosidine biosynthesis	3	27	0.23700768	0.5607175
PWY-5148	acyl-CoA thioesterase pathway	2	15	0.240900868	0.5607175
VALDEG-PWY	valine degradation I	3	28	0.254183553	0.5816064

HOMOSER-THRESYN-PWY	threonine biosynthesis from homoserine	2	16	0.264747435	0.5923499
GLUTSYN-PWY	glutamate biosynthesis I	1	5	0.276429963	0.5923499
PWY-695	abscisic acid biosynthesis	1	5	0.276429963	0.5923499
PWY-43	putrescine biosynthesis II	1	5	0.276429963	0.5923499
PWY1F-FLAVSYN	flavonoid biosynthesis	2	17	0.28860052	0.6087667
PWY-3801	sucrose degradation to ethanol and lactate	14	191	0.306083024	0.6357109
PWY-5153	anthocyanin biosynthesis (delphinidin 3-O-g	1	6	0.321820956	0.6582701
PWY-1121	suberin biosynthesis	2	19	0.33596268	0.6738539
PWY-4021	β-alanine betaine biosynthesis	2	20	0.359317877	0.6738539
OXIDATIVEPENT-PWY	pentose phosphate pathway (oxidative bran	2	20	0.359317877	0.6738539
PWY-922	mevalonate pathway	2	20	0.359317877	0.6738539
GLYSYN-PWY	glycine biosynthesis I	1	7	0.36438028	0.6738539
PWY-5138	fatty acid β-oxidation IV (plant, unsatu	1	7	0.36438028	0.6738539
CAROTENOID-PWY	carotenoid biosynthesis	1	7	0.36438028	0.6738539
PHESYN	phenylalanine biosynthesis I	2	21	0.382369783	0.6975665
PWY-5177	glutaryl-CoA degradation	1	9	0.441695739	0.7644734
CENTFERM-PWY	acetyl CoA fermentation to butyrate	1	9	0.441695739	0.7644734
URSIN-PWY	ureide biosynthesis	1	9	0.441695739	0.7644734
PWY-5125	anthocyanin biosynthesis (pelargonidin 3-O	1	9	0.441695739	0.7644734
SUCSYN-PWY	sucrose biosynthesis	2	25	0.470587023	0.7754715
PWY-981	salicylate biosynthesis	1	10	0.47677136	0.7754715
GLUTAMINEFUM-PWY	glutamine degradation III	1	10	0.47677136	0.7754715
LYSINE-DEG1-PWY	lysine degradation II	1	10	0.47677136	0.7754715
GLUDEG-I-PWY	glutamate degradation III	1	10	0.47677136	0.7754715
FASYN-INITIAL-PWY	fatty acid biosynthesis - initial steps	2	26	0.491465056	0.7898546
PWY-5044	purine degradation	1	11	0.50965557	0.7911966
PWY1F-467	phenylpropanoid biosynthesis, initial reacti	1	11	0.50965557	0.7911966
PWY-702	methionine biosynthesis II	2	27	0.511821646	0.7911966
FASYN-ELONG-PWY	fatty acid elongation -- saturated	2	28	0.531639961	0.7911966
PWY-5269	cardiolipin biosynthesis	1	12	0.540484517	0.7911966
PWY-5386	methylglyoxal degradation I	1	12	0.540484517	0.7911966
PWY-2161	folate polyglutamylation I	1	12	0.540484517	0.7911966
SUCUTIL-PWY	sucrose degradation I	1	12	0.540484517	0.7911966
GLUCONEO-PWY	gluconeogenesis	5	78	0.546305249	0.7911966
P121-PWY	salvage pathways of purine nucleosides	2	29	0.550907286	0.7911966
PWY-621	sucrose degradation III	3	47	0.572702541	0.8138405
PWY-3701	biotin biosynthesis II	1	14	0.596479674	0.8340615
PWY0-163	salvage pathways of pyrimidine ribonucleot	2	32	0.605328884	0.8340615
PWY-3561	choline biosynthesis III	1	15	0.62187816	0.8340615
PHOSLIPSYN2-PWY	phospholipid biosynthesis II	2	34	0.638769482	0.8340615
PWY-2201	folate transformations	1	16	0.645686875	0.8340615
PWY-882	ascorbate biosynthesis	1	16	0.645686875	0.8340615
PWY-842	starch degradation	3	54	0.667973792	0.8340615
PWY-3841	formylTHF biosynthesis II	1	17	0.668004772	0.8340615
P185-PWY	xylulose-monophosphate cycle	1	17	0.668004772	0.8340615
PWY-5136	fatty acid β-oxidation II (plant, saturat	1	17	0.668004772	0.8340615
DTDPRHAMSYN-PWY	dTDP-L-rhamnose biosynthesis I	2	36	0.669959306	0.8340615
TRNA-CHARGING-PWY	tRNA charging pathway	4	72	0.670704669	0.8340615
LIPASYN-PWY	phospholipases	2	37	0.684725145	0.8340615
PWY-3821	galactose degradation II	3	56	0.692189231	0.8340615
P344-PWY	acrylonitrile degradation	1	19	0.70853368	0.8340615
P345-PWY	aldoxime degradation	1	19	0.70853368	0.8340615
PWY-3161	IAA biosynthesis VI (via indole-3-acetamide	1	19	0.70853368	0.8340615
TRPSYN-PWY-1	tryptophan biosynthesis	1	19	0.70853368	0.8340615
ARGDEG-V-PWY	arginine degradation X (arginine monooxyg	1	19	0.70853368	0.8340615
PWY-5461	betanidin degradation	10	181	0.710496853	0.8340615
P1-PWY	salvage pathways of purine and pyrimidine	3	61	0.746900125	0.8692372
PWY-5046	branched-chain α-keto acid dehydrog	1	23	0.775420271	0.8947157
GALACTMETAB-PWY	UDP-galactose biosynthesis (salvage pathw	2	47	0.804395969	0.9191393
CALVIN-PWY	Calvin cycle	3	68	0.810204288	0.9191393
PWY-4041	γ-glutamyl cycle	1	27	0.827027225	0.9304056

FAO-PWY	fatty acid β-oxidation I	1	28	0.837967494	0.9309728
P61-PWY	UDP-glucose conversion	3	73	0.84683135	0.9309728
PWY-5083	NAD/NADH phosphorylation and dephosph	1	29	0.848219645	0.9309728
PWY-622	starch biosynthesis	1	30	0.857826716	0.9339242
ANARESPDON-PWY	respiration (anaerobic)-- electron donors re	1	36	0.904015441	0.9598783
PHOSLIPSYN-PWY	phospholipid biosynthesis I	1	36	0.904015441	0.9598783
AERESPDON-PWY	aerobic respiration -- electron donors reacti	1	36	0.904015441	0.9598783
UDPNACETYLGALSYN-PWY	UDP-<i>N</i>-acetylgalactosamine biosynth	1	37	0.910106838	0.9598783
PWY-1001	cellulose biosynthesis	2	67	0.930476033	0.9659301
PWY-735	jasmonic acid biosynthesis	1	41	0.930861947	0.9659301
PWY-1081	homogalacturonan degradation	5	137	0.939873816	0.9659301
PWY-3781	aerobic respiration -- electron donor II	3	95	0.944464994	0.9659301
LIPAS-PWY	triacylglycerol degradation	3	99	0.95430162	0.968652
GLYCOLYSIS	glycolysis I	2	107	0.992757434	0.9984313
PWY-1042	glycolysis IV (plant cytosol)	1	98	0.998431326	0.9984313

Table S4 (B). RiceCyc Pathways of DE rice genes at 4 hpi

Pathway ID	Pathway Name	Num	Expected Num	p-val	FDR
PWY-5337	stachyose biosynthesis	1	1	0.0066691	0.1030459
PWY-84	resveratrol biosynthesis	1	1	0.0066691	0.1030459
PWY-5036	gibberellin biosynthesis II (early C-3 hydroxylation)	1	2	0.0132963	0.1030459
PWY-5070	gibberellin biosynthesis I (non C-3, non C-13 hydroxylation)	1	2	0.0132963	0.1030459
PWY-5059	pinobanksin biosynthesis	1	3	0.0198816	0.1232659
PWY-5098	chlorophyll <i>a</i> degradation	1	4	0.0264254	0.1308359
PWY-735	jasmonic acid biosynthesis	2	41	0.0295436	0.1308359
PWY-381	nitrate reduction II (assimilatory)	1	8	0.0521903	0.1797667
PWY-5035	gibberellin biosynthesis III (early C-13 hydroxylation)	1	8	0.0521903	0.1797667
PWY-981	salicylate biosynthesis	1	10	0.06483	0.185879
PWY1F-467	phenylpropanoid biosynthesis, initial reactions	1	11	0.07109	0.185879
PWY-1001	cellulose biosynthesis	2	67	0.0719532	0.185879
PWY-5410	13-LOX and 13-HPL pathway	1	15	0.0957363	0.2119875
PWY-5409	divinyl ether biosynthesis II (13-LOX)	1	15	0.0957363	0.2119875
PWY1F-FLAVSYN	flavonoid biosynthesis	1	17	0.1078266	0.2228416
PWY-1121	suberin biosynthesis	1	19	0.1197641	0.2291741
PWY-922	mevalonate pathway	1	20	0.1256761	0.2291741
PWY-5188	tetrapyrrole biosynthesis I	1	33	0.199199	0.3430649
PWY-842	starch degradation	1	54	0.3057667	0.4721583
PWY-5082	methionine degradation III	1	55	0.3104912	0.4721583
PWY66-21	oxidative ethanol degradation I	1	57	0.3198492	0.4721583
PWY-5079	phenylalanine degradation III	1	61	0.3382064	0.4739849
P127-PWY	ethanol fermentation to acetate	1	64	0.3516662	0.4739849
CALVIN-PWY	Calvin cycle	1	68	0.3692108	0.4768973
FERMENTATION-PWY	mixed acid fermentation	1	82	0.4271508	0.5232973
ENTBACSYN-PWY	enterobactin biosynthesis	1	85	0.4388945	0.5232973
PWY-2582	brassinosteroid biosynthesis II	1	92	0.4654184	0.5246002
PWY-3781	aerobic respiration -- electron donor II	1	95	0.4764194	0.5246002
LIPAS-PWY	triacylglycerol degradation	1	99	0.490755	0.5246002
PWY-5461	betanidin degradation	1	181	0.7145214	0.7343191
PWY-3801	sucrose degradation to ethanol and lactate (anaerobic)	1	191	0.7343191	0.7343191

Table S4 (C). RiceCyc Pathways of DE rice genes at 8 hpi

Pathway ID	Pathway Name	Num	Expected Num	p-val	FDR
ETHYL-PWY	ethylene biosynthesis from methionine	2	19	0.0040636	0.0218807
PWY-1001	cellulose biosynthesis	3	67	0.0043761	0.0218807
PWY-622	starch biosynthesis	2	30	0.0100055	0.0333518
PWY-3101	flavonol biosynthesis	1	5	0.0256867	0.0642168
ARGSYN-PWY	arginine biosynthesis I	1	9	0.0457935	0.091587
PWY-5461	betanidin degradation	3	181	0.0624144	0.104024
ARGSYNBSUB-PWY	arginine biosynthesis II (acetyl cycle)	1	18	0.0896333	0.1280475
PWY0-162	de novo biosynthesis of pyrimidine ribonucleotides	1	26	0.1270243	0.1587804
PWY-842	starch degradation	1	54	0.2469581	0.2743979
PWY-3781	aerobic respiration -- electron donor II	1	95	0.3952202	0.3952202

Table S4 (D). RiceCyc Pathways of DE rice genes at 12 hpi

Pathway ID	Pathway Name	Num	Expected Num	p-val	FDR
PWY-2901	cytokinins 9-<i>N</i>-glucoside biosynthesis	4	162	0.0128918	0.1058194
PWY-2881	cytokinins 7-<i>N</i>-glucoside biosynthesis	4	162	0.0128918	0.1058194
PWY-2902	cytokinins-<i>O</i>-glucoside biosynthesis	4	162	0.0128918	0.1058194
PWY-2463	medicarpin biosynthesis	1	4	0.0235154	0.1058194
PWY-2464	maackiain biosynthesis	1	4	0.0235154	0.1058194
PWY-5098	chlorophyll <i>a</i> degradation	1	4	0.0235154	0.1058194
PWY-5409	divinyl ether biosynthesis II (13-LOX)	1	15	0.0855379	0.1845942
PWY-5410	13-LOX and 13-HPL pathway	1	15	0.0855379	0.1845942
PWY1F-FLAVSYN	flavonoid biosynthesis	1	17	0.0964101	0.1845942
PWY-2582	brassinosteroid biosynthesis II	2	92	0.1012374	0.1845942
PWY-3161	IAA biosynthesis VI (via indole-3-acetamide)	1	19	0.107161	0.1845942
ARGDEG-V-PWY	arginine degradation X (arginine monooxygenase pathway)	1	19	0.107161	0.1845942
P344-PWY	acrylonitrile degradation	1	19	0.107161	0.1845942
P345-PWY	aldoxime degradation	1	19	0.107161	0.1845942
CYSTSYN-PWY	cysteine biosynthesis I	1	21	0.117792	0.1845942
PWY-2941	lysine biosynthesis II	1	22	0.1230628	0.1845942
PWY-5097	lysine biosynthesis VI	1	22	0.1230628	0.1845942
DAPLYSINESYN-PWY	lysine biosynthesis I	1	22	0.1230628	0.1845942
PWY-735	jasmonic acid biosynthesis	1	41	0.21777	0.3094626
PWY-1001	cellulose biosynthesis	1	67	0.3319106	0.4480794
GLUCONEO-PWY	gluconeogenesis	1	78	0.3753319	0.4825696
ENTBACSYN-PWY	enterobactin biosynthesis	1	85	0.4015707	0.4928368
PWY-3781	aerobic respiration -- electron donor II	1	95	0.4372671	0.5035279
PWY-1042	glycolysis IV (plant cytosol)	1	98	0.4475804	0.5035279
GLYCOLYSIS	glycolysis I	1	107	0.4774672	0.5156646
PWY-5461	betanidin degradation	1	181	0.6717143	0.6920232
PWY-3801	sucrose degradation to ethanol and lactate (anaerobic)	1	191	0.6920232	0.6920232

Table S4 (E). RiceCyc Pathways of DE rice genes at 24 hpi

Pathway ID	Pathway Name	Num	Expected Num	p-val	FDR
PWY-2902	cytokinins-<i>O</i>-glucoside biosynthesis	11	162	0.001764	0.0336095
PWY-2881	cytokinins 7-<i>N</i>-glucoside biosynthesis	11	162	0.001764	0.0336095
PWY-2901	cytokinins 9-<i>N</i>-glucoside biosynthesis	11	162	0.001764	0.0336095
PWY-102	gibberellin inactivation	2	3	0.0017925	0.0336095
PWY-2463	medicarpin biosynthesis	2	4	0.0035275	0.0440939
PWY-2464	maackiain biosynthesis	2	4	0.0035275	0.0440939
PWY-5338	galactosylcyclitol biosynthesis	2	6	0.0085389	0.0914883
PWY-581	IAA biosynthesis I	4	35	0.0101798	0.0954357
PWY-84	resveratrol biosynthesis	1	1	0.024824	0.1861801
PWY0-1182	trehalose degradation II (high osmolarity)	1	1	0.024824	0.1861801
PWY-4361	methionine salvage pathway	2	14	0.045587	0.2933862
PWY-5188	tetrapyrrole biosynthesis I	3	33	0.0469418	0.2933862
PWY-5059	pinobanksin biosynthesis	1	3	0.0726649	0.3710814
PWY-2582	brassinosteroid biosynthesis II	5	92	0.075914	0.3710814
TRESYN-PWY	trehalose biosynthesis I	2	19	0.079164	0.3710814
ETHYL-PWY	ethylene biosynthesis from methionine	2	19	0.079164	0.3710814
PWY-5098	chlorophyll <i>a</i> degradation	1	4	0.0957107	0.422253
PWY-842	starch degradation	3	54	0.1486877	0.6183298
SAM-PWY	S-adenosylmethionine biosynthesis	1	7	0.1615159	0.6183298
PWY-5084	2-keto glutarate dehydrogenase complex	1	8	0.1823845	0.6183298
CHLOROPHYLL-SYN	chlorophyllide <i>a</i> biosynthesis	2	32	0.1877232	0.6183298
PWY-5041	<i>S</i>-adenosyl-L-methionine cycle	1	9	0.2027414	0.6183298
PWY-981	salicylate biosynthesis	1	10	0.2225987	0.6183298
PWY66-162	oxidative ethanol degradation III	1	10	0.2225987	0.6183298
SALVADEHYPOX-PWY	salvage pathways of adenine, hypoxanthine, and their nucleosides	1	10	0.2225987	0.6183298
GLUDEG-I-PWY	glutamate degradation III	1	10	0.2225987	0.6183298
LYSINE-DEG1-PWY	lysine degradation II	1	10	0.2225987	0.6183298
PWY1F-467	phenylpropanoid biosynthesis, initial reactions	1	11	0.2419687	0.6257743
PWY-401	glycolipid biosynthesis	1	12	0.2608631	0.6257743
GDPRHAMSYN-PWY	GDP-D-rhamnose biosynthesis	1	12	0.2608631	0.6257743
PWY-735	jasmonic acid biosynthesis	2	41	0.2708201	0.6257743
PWY-66	GDP-L-fucose biosynthesis I (from GDP-D-mannose)	1	13	0.2792934	0.6257743
PWY-3701	biotin biosynthesis II	1	14	0.2972708	0.6257743
DETOX1-PWY	removal of superoxide radicals	1	14	0.2972708	0.6257743
HEME-BIOSYNTHESIS-II	heme biosynthesis II	1	15	0.3148063	0.6257743
PWY-5409	divinyl ether biosynthesis II (13-LOX)	1	15	0.3148063	0.6257743
PWY-5410	13-LOX and 13-HPL pathway	1	15	0.3148063	0.6257743
P185-PWY	xylulose-monophosphate cycle	1	17	0.3485942	0.6257743
PWY1F-FLAVSYN	flavonoid biosynthesis	1	17	0.3485942	0.6257743
ENTBACSYN-PWY	enterobactin biosynthesis	3	85	0.353796	0.6257743
PWY-1121	suberin biosynthesis	1	19	0.3807396	0.6257743
OXIDATIVEPENT-PWY	pentose phosphate pathway (oxidative branch)	1	20	0.3962211	0.6257743
PWY-922	mevalonate pathway	1	20	0.3962211	0.6257743
PWY-5082	methionine degradation III	2	55	0.398968	0.6257743
CYSTSYN-PWY	cysteine biosynthesis I	1	21	0.4113212	0.6257743
PWY66-21	oxidative ethanol degradation I	2	57	0.4165624	0.6257743
PWY-3781	aerobic respiration -- electron donor II	3	95	0.4221168	0.6257743
PWY-5097	lysine biosynthesis VI	1	22	0.4260492	0.6257743
PWY-2941	lysine biosynthesis II	1	22	0.4260492	0.6257743
DAPLYSINESYN-PWY	lysine biosynthesis I	1	22	0.4260492	0.6257743
PWY-5046	branched-chain α-keto acid dehydrogenase complex	1	23	0.440414	0.6257743
PWY-5043	salvage pathways of purine nucleosides II (plant)	1	23	0.440414	0.6257743
PWY-1042	glycolysis IV (plant cytosol)	3	98	0.4422138	0.6257743
PWY-5079	phenylalanine degradation III	2	61	0.4509779	0.6263582
P127-PWY	ethanol fermentation to acetate	2	64	0.4760489	0.6285946
PWY-4041	γ-glutamyl cycle	1	27	0.4944156	0.6285946
PWY-1001	cellulose biosynthesis	2	67	0.5004357	0.6285946
GLYCOLYSIS	glycolysis I	3	107	0.5008274	0.6285946
VALDEG-PWY	valine degradation I	1	28	0.507093	0.6285946
CALVIN-PWY	Calvin cycle	2	68	0.5084065	0.6285946
P121-PWY	salvage pathways of purine nucleosides	1	29	0.5194572	0.6285946
PWY-3801	sucrose degradation to ethanol and lactate (anaerobic)	5	191	0.5196382	0.6285946
PWY-361	phenylpropanoid biosynthesis	1	31	0.5432762	0.6467573
GLUCONEO-PWY	gluconeogenesis	2	78	0.5835392	0.683835
UDPNACETYLGALSYN-PWY	UDP-<i>N</i>-acetylgalactosamine biosynthesis	1	37	0.6079713	0.6841707
LIPASYN-PWY	phospholipases	1	37	0.6079713	0.6841707
FERMENTATION-PWY	mixed acid fermentation	2	82	0.6111925	0.6841707
GALACTMETAB-PWY	UDP-galactose biosynthesis (salvage pathway from galactose using UDP-glucose)	1	47	0.6963167	0.7679963
PWY-4302	aerobic respiration -- electron donor III	1	56	0.7588701	0.8130751
PWY-3821	galactose degradation II	1	56	0.7588701	0.8130751

P1-PWY	salvage pathways of purine and pyrimidine nucleotides	1	61	0.7879434	0.8323346
PWY-5461	betanidin degradation	3	181	0.8387461	0.8675027
P61-PWY	UDP-glucose conversion	1	73	0.8443693	0.8675027
PWY-1081	homogalacturonan degradation	2	137	0.8635829	0.8752529
LIPAS-PWY	triacylglycerol degradation	1	99	0.9207785	0.9207785

Table S4 (F). RiceCyc Pathways of DE rice genes at 48 hpi

Pathway ID	Pathway Name	Num	Expected Num	p-val	FDR
PWY-2463	medicarpin biosynthesis	2	4	0.0003428	0.0070278
PWY-2464	maackiain biosynthesis	2	4	0.0003428	0.0070278
PWY-84	resveratrol biosynthesis	1	1	0.0077807	0.1063357
PWY-5344	homocysteine biosynthesis	1	2	0.0155036	0.1589123
PWY-5059	pinobanksin biosynthesis	1	3	0.0231694	0.1899887
PWY-2582	brassinosteroid biosynthesis II	3	92	0.0326797	0.2233114
PWY-981	salicylate biosynthesis	1	10	0.0752593	0.3063017
PWY1F-467	phenylpropanoid biosynthesis, initial reactions	1	11	0.0824812	0.3063017
THRDLCTCAT-PWY	threonine degradation III (to methylglyoxal)	1	12	0.0896493	0.3063017
PWY-5448	aminopropanol biosynthesis	1	12	0.0896493	0.3063017
PWY-2261	ascorbate glutathione cycle	1	12	0.0896493	0.3063017
THREONINE-DEG2-PWY	threonine degradation II	1	12	0.0896493	0.3063017
PWY-5409	divinyl ether biosynthesis II (13-LOX)	1	15	0.110835	0.313121
PWY-5410	13-LOX and 13-HPL pathway	1	15	0.110835	0.313121
PWY1F-FLAVSYN	flavonoid biosynthesis	1	17	0.1246971	0.313121
PWY-2541	sterol biosynthesis	1	18	0.1315507	0.313121
PWY-1121	suberin biosynthesis	1	19	0.1383531	0.313121
PWY-4021	β-alanine betaine biosynthesis	1	20	0.1451048	0.313121
PWY-922	mevalonate pathway	1	20	0.1451048	0.313121
PWY-4041	γ-glutamyl cycle	1	27	0.1909753	0.3914995
P121-PWY	salvage pathways of purine nucleosides	1	29	0.2036444	0.3975915
PWY-361	phenylpropanoid biosynthesis	1	31	0.2161244	0.4027774
DTDPRHAMSYN-PWY	dTDP-L-rhamnose biosynthesis I	1	36	0.2465147	0.43128
UDPNACETYLGALSYN-PWY	UDP-<i>N</i>-acetylgalactosamine biosynthesis	1	37	0.2524566	0.43128
PWY-841	purine nucleotides <i>de novo</i> biosynthesis II	1	39	0.2642067	0.433299
PWY-735	jasmonic acid biosynthesis	1	41	0.2757808	0.4348851
GALACTMETAB-PWY	UDP-galactose biosynthesis (salvage pathway from galactose using UDP-glucose)	1	47	0.3094718	0.4641049
PWY-3821	galactose degradation II	1	56	0.3572232	0.4641049
DENOVOPURINE2-PWY	purine nucleotides <i>de novo</i> biosynthesis I	1	56	0.3572232	0.4641049
PWY-2902	cytokinins-<i>O</i>-glucoside biosynthesis	2	162	0.3622282	0.4641049
PWY-2901	cytokinins 9-<i>N</i>-glucoside biosynthesis	2	162	0.3622282	0.4641049
PWY-2881	cytokinins 7-<i>N</i>-glucoside biosynthesis	2	162	0.3622282	0.4641049
PWY-1001	cellulose biosynthesis	1	67	0.4113212	0.5016689
CALVIN-PWY	Calvin cycle	1	68	0.4160181	0.5016689
P61-PWY	UDP-glucose conversion	1	73	0.4389722	0.5142246
ENTBACSYN-PWY	enterobactin biosynthesis	1	85	0.4906079	0.5587479
PWY-3781	aerobic respiration -- electron donor II	1	95	0.5301483	0.5841729
PWY-1042	glycolysis IV (plant cytosol)	1	98	0.5414285	0.5841729
GLYCOLYSIS	glycolysis I	1	107	0.5737423	0.603165
PWY-5461	betanidin degradation	1	181	0.7685443	0.7871711
PWY-3801	sucrose degradation to ethanol and lactate (anaerobic)	1	191	0.7871711	0.7871711

Table S4 (G). RiceCyc Pathways of DE rice genes at 72 hpi

Pathway ID	Pathway Name	Num	Expected Num	p-val	FDR
PWY-2902	cytokinins-<i>O</i>-glucoside biosynthesis	12	162	0.0007323	0.0190406
PWY-2901	cytokinins 9-<i>N</i>-glucoside biosynthesis	12	162	0.0007323	0.0190406
PWY-2881	cytokinins 7-<i>N</i>-glucoside biosynthesis	12	162	0.0007323	0.0190406
PWY-2463	medicarpin biosynthesis	2	4	0.0038472	0.0600161
PWY-2464	maackiain biosynthesis	2	4	0.0038472	0.0600161
PWY-3162	tryptophan degradation V (side chain pathway)	2	5	0.0063046	0.0702513
PWY-3641	carnitine degradation III	2	5	0.0063046	0.0702513
PWY-5147	stearoyl-ACP desaturation pathway	2	8	0.0167841	0.1636446
PWY-2261	ascorbate glutathione cycle	2	12	0.0370047	0.3072467
PWY-822	fructan biosynthesis	1	2	0.0512078	0.3072467
PWY-862	fructan degradation	1	2	0.0512078	0.3072467
PWY-5036	gibberellin biosynthesis II (early C-3 hydroxylation)	1	2	0.0512078	0.3072467
PWY-5070	gibberellin biosynthesis I (non C-3, non C-13 hydroxylation)	1	2	0.0512078	0.3072467
PWY-581	IAA biosynthesis I	3	35	0.060538	0.3372834
PWY-5059	pinobanksin biosynthesis	1	3	0.0758335	0.3696881
ASPARAGINESYN-PWY	asparagine biosynthesis III	1	3	0.0758335	0.3696881
PWY-2582	brassinosteroid biosynthesis II	5	92	0.0879648	0.4036033
PWY-1001	cellulose biosynthesis	4	67	0.0938488	0.4066783
PWY-1581	plastoquinone biosynthesis	1	4	0.0998289	0.4098238
PWY-1422	vitamin E biosynthesis	1	5	0.1232099	0.4805188
PWY-5074	mevalonate degradation	1	6	0.1459922	0.5422566
FASYN-ELONG-PWY	fatty acid elongation -- saturated	2	28	0.1631013	0.5782682
PWY-5035	gibberellin biosynthesis III (early C-13 hydroxylation)	1	8	0.1898202	0.5993384
ASPARAGINE-BIOSYNTHESIS	asparagine biosynthesis I	1	8	0.1898202	0.5993384
PWY-5188	tetrapyrrole biosynthesis I	2	33	0.2103084	0.5993384
PWY-5473	hydroxycinnamic acid serotonin amides biosynthesis	1	10	0.2314294	0.5993384
GLUDEG-I-PWY	glutamate degradation III	1	10	0.2314294	0.5993384
PWY-841	purine nucleotides <i>de novo</i> biosynthesis II	2	39	0.2684558	0.5993384
SUCUTIL-PWY	sucrose degradation I	1	12	0.2709307	0.5993384
PWY-282	epicuticular wax biosynthesis	1	12	0.2709307	0.5993384
NONMEVIPP-PWY	methylerythritol phosphate pathway	1	12	0.2709307	0.5993384
PWY-5386	methylglyoxal degradation I	1	12	0.2709307	0.5993384
THRDLCTCAT-PWY	threonine degradation III (to methylglyoxal)	1	12	0.2709307	0.5993384
PWY-5448	aminopropanol biosynthesis	1	12	0.2709307	0.5993384
THREONINE-DEG2-PWY	threonine degradation II	1	12	0.2709307	0.5993384
P61-PWY	UDP-glucose conversion	3	73	0.2935548	0.5993384
TYRFUMCAT-PWY	tyrosine degradation I	1	14	0.3084292	0.5993384
PWY-3701	biotin biosynthesis II	1	14	0.3084292	0.5993384
CYANCAT-PWY	cyanate degradation	1	15	0.326459	0.5993384
HYDROXYPRODEG-PWY	4-hydroxyproline degradation I	1	15	0.326459	0.5993384
PWY-5461	betanidin degradation	6	181	0.3277325	0.5993384
PROUT-PWY	proline degradation I	1	16	0.3440253	0.5993384
PWY-4561	proline degradation II	1	16	0.3440253	0.5993384
HOMOSER-THRESYN-PWY	threonine biosynthesis from homoserine	1	16	0.3440253	0.5993384
ARGASEDEG-PWY	arginine degradation I (arginase pathway)	1	17	0.3611398	0.5993384
PWY1F-FLAVSYN	flavonoid biosynthesis	1	17	0.3611398	0.5993384
P185-PWY	xylulose-monophosphate cycle	1	17	0.3611398	0.5993384
ENTBACSYN-PWY	enterobactin biosynthesis	3	85	0.3799715	0.6093544
TRESYN-PWY	trehalose biosynthesis I	1	19	0.3940591	0.6093544
PWY-1121	suberin biosynthesis	1	19	0.3940591	0.6093544
PWY-4021	β-alanine betaine biosynthesis	1	20	0.4098859	0.6093544
PWY-922	mevalonate pathway	1	20	0.4098859	0.6093544
PWY-5082	methionine degradation III	2	55	0.4206126	0.6093544
PWY-5121	geranylgeranyldiphosphate biosynthesis II (plastidic)	1	21	0.4253051	0.6093544
DENOVOPURINE2-PWY	purine nucleotides <i>de novo</i> biosynthesis I	2	56	0.429673	0.6093544
PWY66-21	oxidative ethanol degradation I	2	57	0.4386607	0.6109917
PWY-3781	aerobic respiration -- electron donor II	3	95	0.4505092	0.6164863
PWY-5079	phenylalanine degradation III	2	61	0.4738409	0.6372343
FASYN-INITIAL-PWY	fatty acid biosynthesis - initial steps	1	26	0.4966398	0.6491736
P127-PWY	ethanol fermentation to acetate	2	64	0.4993643	0.6491736
PWY-5290	secologanin and strictosidine biosynthesis	1	27	0.5098217	0.6519032

PWY-5083	NAD/NADH phosphorylation and dephosphorylation	1	29	0.535173	0.6625951
P121-PWY	salvage pathways of purine nucleosides	1	29	0.535173	0.6625951
PWY-361	phenylpropanoid biosynthesis	1	31	0.5592309	0.6815626
PHOSLIPSYN2-PWY	phospholipid biosynthesis II	1	34	0.5930342	0.711641
ANARESPDON-PWY	respiration (anaerobic)-- electron donors reaction list	1	36	0.6141364	0.7149647
AERESPDON-PWY	aerobic respiration -- electron donors reaction list	1	36	0.6141364	0.7149647
FERMENTATION-PWY	mixed acid fermentation	2	82	0.6353163	0.7287452
PWY-621	sucrose degradation III	1	47	0.7123026	0.8052116
PWY-4302	aerobic respiration -- electron donor III	1	56	0.7739418	0.8623923
CALVIN-PWY	Calvin cycle	1	68	0.8363049	0.9187575
TRNA-CHARGING-PWY	tRNA charging pathway	1	72	0.853052	0.9191815
GLUCONEO-PWY	gluconeogenesis	1	78	0.8750548	0.9191815
PWY-1081	homogalacturonan degradation	2	137	0.8795974	0.9191815
PWY-3801	sucrose degradation to ethanol and lactate (anaerobic)	3	191	0.8838284	0.9191815
PWY-1042	glycolysis IV (plant cytosol)	1	98	0.9274362	0.9414591
LIPAS-PWY	triacylglycerol degradation	1	99	0.9293891	0.9414591
GLYCOLYSIS	glycolysis I	1	107	0.943256	0.943256

Table S4 (H). RiceCyc Pathways of DE rice genes at 96 hpi

Pathway ID	Pathway Name	Num	Expected Num	p-val	FDR
PWY-2902	cytokinins-<i>O</i>-glucoside biosynthesis	18	162	2.08E-06	4.43E-05
PWY-2901	cytokinins 9-<i>N</i>-glucoside biosynthesis	18	162	2.08E-06	4.43E-05
PWY-2881	cytokinins 7-<i>N</i>-glucoside biosynthesis	18	162	2.08E-06	4.43E-05
PWY-735	jasmonic acid biosynthesis	9	41	3.73E-06	5.96E-05
PWY-5409	divinyl ether biosynthesis II (13-LOX)	4	15	0.0010478	0.0111768
PWY-5410	13-LOX and 13-HPL pathway	4	15	0.0010478	0.0111768
PWY-581	IAA biosynthesis I	4	35	0.0246134	0.2198775
PWY-5082	methionine degradation III	5	55	0.0304079	0.2198775
PWY66-21	oxidative ethanol degradation I	5	57	0.0348176	0.2198775
PWY-5046	branched-chain α-keto acid dehydrogenase complex	3	23	0.0359619	0.2198775
PWY-5473	hydroxycinnamic acid serotonin amides biosynthesis	2	10	0.03907	0.2198775
PWY-5079	phenylalanine degradation III	5	61	0.0447799	0.2198775
P127-PWY	ethanol fermentation to acetate	5	64	0.0532704	0.2198775
THRDLCTCAT-PWY	threonine degradation III (to methylglyoxal)	2	12	0.0549694	0.2198775
PWY-5448	aminopropanol biosynthesis	2	12	0.0549694	0.2198775
THREONINE-DEG2-PWY	threonine degradation II	2	12	0.0549694	0.2198775
CHLOROPHYLL-SYN	chlorophyllide <i>a</i> biosynthesis	3	32	0.082013	0.3087547
LIPASYN-PWY	phospholipases	3	37	0.1147706	0.3759191
FERMENTATION-PWY	mixed acid fermentation	5	82	0.1223958	0.3759191
PWY4KF-48	momilactone biosynthesis	1	4	0.1229003	0.3759191
TRPSYN-PWY-1	tryptophan biosynthesis	2	19	0.1233485	0.3759191
ENTBACSYN-PWY	enterobactin biosynthesis	5	85	0.1367601	0.3978475
PWY-3101	flavonol biosynthesis	1	5	0.1512148	0.4207717
PWY-3801	sucrose degradation to ethanol and lactate (anaerobic)	9	191	0.1584469	0.4225251
SAM-PWY	S-adenosylmethionine biosynthesis	1	7	0.2051608	0.5094585
PWY-4041	γ-glutamyl cycle	2	27	0.2155548	0.5094585
PWY-5290	secologanin and strictosidine biosynthesis	2	27	0.2155548	0.5094585
PWY-381	nitrate reduction II (assimilatory)	1	8	0.2308484	0.5094585
PWY-5035	gibberellin biosynthesis III (early C-13 hydroxylation)	1	8	0.2308484	0.5094585
PWY-842	starch degradation	3	54	0.2515696	0.5279278
PWY-5041	<i>S</i>-adenosyl-L-methionine cycle	1	9	0.2557151	0.5279278
PWY-5188	tetrapyrrole biosynthesis I	2	33	0.2880937	0.5761874
SUCUTIL-PWY	sucrose degradation I	1	12	0.3256448	0.6129785
PWY-2261	ascorbate glutathione cycle	1	12	0.3256448	0.6129785
RIBOKIN-PWY	ribose degradation	1	13	0.3474792	0.6353905
PWY-4361	methionine salvage pathway	1	14	0.3686144	0.6553145
HEME-BIOSYNTHESIS-II	heme biosynthesis II	1	15	0.3890727	0.6729907
ARO-PWY	chorismate biosynthesis	1	16	0.4088755	0.6886324
PWY1F-FLAVSYN	flavonoid biosynthesis	1	17	0.4280435	0.7024304
PWY-2541	sterol biosynthesis	1	18	0.4465969	0.7078936
PWY-1121	suberin biosynthesis	1	19	0.4645552	0.7078936
ETHYL-PWY	ethylene biosynthesis from methionine	1	19	0.4645552	0.7078936
PWY-922	mevalonate pathway	1	20	0.4819372	0.7173018
PWY-5121	geranylgeranyldiphosphate biosynthesis II (plastidic)	1	21	0.4987612	0.7254708
PWY-5043	salvage pathways of purine nucleosides II (plant)	1	23	0.5308055	0.7433751
PWY-5461	betanidin degradation	6	181	0.5343009	0.7433751
PWY-181	photorespiration	1	26	0.5751121	0.7831313
PWY-1042	glycolysis IV (plant cytosol)	3	98	0.619977	0.8240743
PWY-361	phenylpropanoid biosynthesis	1	31	0.6399461	0.8240743
PWY-1001	cellulose biosynthesis	2	67	0.6438081	0.8240743
GLYCOLYSIS	glycolysis I	3	107	0.679601	0.8354943
P61-PWY	UDP-glucose conversion	2	73	0.6902572	0.8354943
DTDPRHAMSYN-PWY	dTDP-L-rhamnose biosynthesis I	1	36	0.6949834	0.8354943
UDPNACETYLGALSYN-PWY	UDP-<i>N</i>-acetylgalactosamine biosynthesis	1	37	0.7049483	0.8354943
PWY-841	purine nucleotides <i>de novo</i> biosynthesis II	1	39	0.7239225	0.8423826
GALACTMETAB-PWY	UDP-galactose biosynthesis (salvage pathway from galactose using UDP-glucose)	1	47	0.7884849	0.8706162
PWY-621	sucrose degradation III	1	47	0.7884849	0.8706162
PWY-2582	brassinosteroid biosynthesis II	2	92	0.8056648	0.8706162
PWY-3781	aerobic respiration -- electron donor II	2	95	0.819969	0.8706162
PWY-1081	homogalacturonan degradation	3	137	0.8286778	0.8706162
PWY-3821	galactose degradation II	1	56	0.8434094	0.8706162
DENOVOPURINE2-PWY	purine nucleotides <i>de novo</i> biosynthesis I	1	56	0.8434094	0.8706162
CALVIN-PWY	Calvin cycle	1	68	0.8952969	0.909508
GLUCONEO-PWY	gluconeogenesis	1	78	0.9252403	0.9252403

Table S5 (A). Significantly enriched (FDR<0.1) GO Annotations of DE rice genes S vs Mock

GO ID	GO Category	Term Name	GO Level	Num	List Total	Expected Num	Pop Total	p-val	FDR
GO:0009579	cellular_component	thylakoid	3	228	4338	560	20718	3.98E-27	9.96E-26
GO:0009536	cellular_component	plastid	4	916	4338	3318	20718	7.33E-24	9.17E-23
GO:0015979	biological_process	photosynthesis	3	98	5080	234	24187	3.48E-13	1.57E-11
GO:0009628	biological_process	response to abiotic stimulus	2	549	5080	2197	24187	1.33E-06	2.99E-05
GO:0016020	cellular_component	membrane	1	882	4338	3729	20718	4.77E-06	3.98E-05
GO:0006091	biological_process	generation of precursor metabolites and energy	3	104	5080	330	24187	4.50E-06	6.75E-05
GO:0009607	biological_process	response to biotic stimulus	2	277	5080	1076	24187	7.76E-05	0.000873413
GO:0003824	molecular_function	catalytic activity	1	861	4965	3685	23524	0.00015695	0.00408061
GO:0006950	biological_process	response to stress	2	829	5080	3617	24187	0.00125296	0.011276636
GO:0003682	molecular_function	chromatin binding	2	15	4965	37	23524	0.00571233	0.074260314
GO:0019748	biological_process	secondary metabolic process	3	124	5080	488	24187	0.01029494	0.077212051
GO:0040029	biological_process	regulation of gene expression, epigenetic	6	38	5080	128	24187	0.01270356	0.081665729

Table S5 (B). Significantly enriched (FDR<0.1) GO Annotations of DE rice genes in R vs Mock

GO ID	GO Category	Term Name	GO Level	Num	List Total	Expected Num	Pop Total	p-val	FDR
GO:0009579	cellular_component	thylakoid	3	184	3803	560	20718	5.60E-17	1.40E-15
GO:0015979	biological_process	photosynthesis	3	80	4481	234	24187	7.79E-09	3.43E-07
GO:0009536	cellular_component	plastid	4	705	3803	3318	20718	2.08E-06	2.60E-05
GO:0016020	cellular_component	membrane	1	780	3803	3729	20718	5.85E-06	4.88E-05
GO:0019748	biological_process	secondary metabolic process	3	130	4481	488	24187	5.35E-06	0.00011762
GO:0009628	biological_process	response to abiotic stimulus	2	479	4481	2197	24187	2.63E-05	0.000385931
GO:0009607	biological_process	response to biotic stimulus	2	250	4481	1076	24187	4.39E-05	0.000483058
GO:0008152	biological_process	metabolic process	1	1475	4481	7383	24187	6.80E-05	0.000598656
GO:0006950	biological_process	response to stress	2	727	4481	3617	24187	0.00468603	0.034364197
GO:0005215	molecular_function	transporter activity	1	317	4411	1456	23524	0.00150351	0.039091385
GO:0006091	biological_process	generation of precursor metabolites and energy	3	79	4481	330	24187	0.0079408	0.04991358

Table S5 (C). Significantly enriched (FDR<0.1) GO Annotations of DE rice genes in S vs R uninfected

GO ID	GO Category	Term Name	GO Level	Num	List Total	Expected Num	Pop Total	p-val	FDR
GO:0009579	cellular_component	thylakoid	3	91	1232	560	20718	1.22E-18	4.99E-17
GO:0009536	cellular_component	plastid	4	291	1232	3318	20718	6.05E-13	1.24E-11
GO:0030529	cellular_component	ribonucleoprotein complex	2	61	1232	480	20718	1.70E-08	1.39E-07
GO:0005840	cellular_component	ribosome	3	61	1232	480	20718	1.70E-08	1.39E-07
GO:0032991	cellular_component	macromolecular complex	1	61	1232	480	20718	1.70E-08	1.39E-07
GO:0015979	biological_process	photosynthesis	3	39	1412	234	24187	2.96E-09	2.82E-07
GO:0044444	cellular_component	cytoplasmic part	3	541	1232	7703	20718	3.45E-07	2.35E-06
GO:0043232	cellular_component	intracellular non-membrane-bounded organelle	3	92	1232	922	20718	6.78E-07	3.47E-06
GO:0043228	cellular_component	non-membrane-bounded organelle	2	92	1232	922	20718	6.78E-07	3.47E-06
GO:1901576	biological_process	organic substance biosynthetic process	3	69	1412	668	24187	3.07E-06	4.86E-05
GO:0044249	biological_process	cellular biosynthetic process	3	69	1412	668	24187	3.07E-06	4.86E-05
GO:0034645	biological_process	cellular macromolecule biosynthetic process	4	69	1412	668	24187	3.07E-06	4.86E-05
GO:0009059	biological_process	macromolecule biosynthetic process	4	69	1412	668	24187	3.07E-06	4.86E-05
GO:0006412	biological_process	translation	5	69	1412	668	24187	3.07E-06	4.86E-05
GO:0005198	molecular_function	structural molecule activity	1	59	1411	515	23524	1.43E-06	5.16E-05
GO:0044424	cellular_component	intracellular part	2	683	1232	10323	20718	2.72E-05	0.0001237
GO:0044464	cellular_component	cell part	1	842	1232	13103	20718	6.32E-05	0.0002589
GO:0043231	cellular_component	intracellular membrane-bounded organelle	3	549	1232	8205	20718	0.0001474	0.0005036
GO:0043227	cellular_component	membrane-bounded organelle	2	549	1232	8205	20718	0.0001474	0.0005036
GO:0043229	cellular_component	intracellular organelle	2	574	1232	8633	20718	0.0001793	0.0005251
GO:0043226	cellular_component	organelle	1	574	1232	8633	20718	0.0001793	0.0005251
GO:0016020	cellular_component	membrane	1	409	1232	5987	20718	0.0003864	0.001056
GO:0005739	cellular_component	mitochondrion	4	93	1232	1133	20718	0.0009422	0.0024145
GO:0009628	biological_process	response to abiotic stimulus	2	159	1412	2197	24187	0.0024766	0.0336104
GO:0005829	cellular_component	cytosol	4	157	1232	2285	20718	0.0282816	0.0634857
GO:0044428	cellular_component	nuclear part	3	46	1232	583	20718	0.0309686	0.0634857
GO:0044422	cellular_component	organelle part	1	46	1232	583	20718	0.0309686	0.0634857
GO:0044446	cellular_component	intracellular organelle part	2	46	1232	583	20718	0.0309686	0.0634857

Table S5 (D). Significantly enriched (FDR<0.1) GO Annotations of DE rice genes in S vs R infected

GO ID	GO Category	Term Name	GO Level	Num	List Total	Expected Num	Pop Total	p-val	FDR
GO:0008152	biological_process	metabolic process	1	553	1510	7383	24187	9.59E-08	4.12E-06
GO:0019825	molecular_function	oxygen binding	2	41	1471	345	23524	6.07E-05	0.0015785
GO:0016740	molecular_function	transferase activity	2	160	1471	1934	23524	0.0001357	0.0017639
GO:0019748	biological_process	secondary metabolic process	3	52	1510	488	24187	0.0001206	0.0023843
GO:0009607	biological_process	response to biotic stimulus	2	97	1510	1076	24187	0.0001663	0.0023843
GO:0003824	molecular_function	catalytic activity	1	278	1471	3685	23524	0.0003227	0.0027967
GO:0005215	molecular_function	transporter activity	1	120	1471	1456	23524	0.0010864	0.0070613
GO:0006950	biological_process	response to stress	2	270	1510	3617	24187	0.000714	0.0076756
GO:0009628	biological_process	response to abiotic stimulus	2	172	1510	2197	24187	0.0010149	0.0087284
GO:0005623	cellular_component	cell	1	209	1292	2705	20718	0.0004702	0.0112853
GO:0016020	cellular_component	membrane	1	273	1292	3729	20718	0.001657	0.0198835
GO:0009719	biological_process	response to endogenous stimulus	2	116	1510	1492	24187	0.0080932	0.0580012
GO:0008289	molecular_function	lipid binding	2	27	1471	271	23524	0.0115024	0.0598124
GO:0005488	molecular_function	binding	1	267	1471	3793	23524	0.016912	0.0732854

Table S6. Primers used in RT-PCR validation of gene expression

Gene ID or Symbol	Forward	Reverse
LOC_Os02g18140	TCGGTACTTTGAAGACCTTTTTCT	TGCAACAGGCAGTAGGTTCTT
LOC_Os11g36180	GGCTATGCAGCACCAGAGTAT	GGCCCAGTTCAACGTACTGA
LOC_Os11g35710	CCTTGTCCGGTACGTTGGTT	GTTCGTCGACCGCAACAAAG
LOC_Os11g36160	GGTTACGCAGCACCAGAGTA	AGTATCGGTTGGTCGCTTTCC
LOC_Os05g08480	TCAGTGGCATCAGAAGTCGC	TGCCATTGTCGTCTACTACAGTTA
LOC_Os03g55010	GAGGAGAAGGGAGATCGGCA	GTAGGCGTCGATGAGCTTGT
LOC_Os05g45200	GCTACCGGCCTACTTCTTCC	CTGGAACGTGTTGACGAGGA
LOC_Os01g45140	GCGTTACACCGACCAGTTCT	CGATGGTCAATGAACGACGC
LOC_Os01g01650	GCCGTGCTCACCAAAATCAA	CTCCCCTCTACAGTAGGCCG
LOC_Os01g01660	CCGTTGAACATCGTGCTGTC	GACACTCACTCTCACCGACG
LOC_Os07g46870	ATCAGGTCGATTCTGCTCTGC	GACGACTAAACCAGCTGTCCA
LOC_Os11g30560	CATCGGCGAGCATCTAGTGT	ACGTTGTGGAAGGCGGTTAT
LOC_Os04g33240	TGGCATCTTCAAGGACTGAGAC	GTCGATCGTGACACAAGCCT
Xa21 expression	GCTCTGCCCTTCAAGCAGT	CTTGAAAGAGAGCAGCGCGA
U1/I1	CGATCGGTATAACAGCAAAAC	TCTGATCATGCATGTTCTGTG
